# In-Stent Ulceration: An Unusual Pathology

**DOI:** 10.1155/2014/893143

**Published:** 2014-03-04

**Authors:** Jagadeesh Kumar Kalavakunta, Shravan Gangula, Vishal Gupta

**Affiliations:** ^1^Department of Cardiology, Michigan State University, East Lansing, MI 48824, USA; ^2^Department of Cardiology, Borgess Medical Center, 804 Service Road A205, Kalamazoo, MI 49048, USA

## Abstract

In-stent restenosis occurs in 10–60% of cases undergoing interventional therapy. Many mechanisms explain the reason for in-stent restenosis, but restenosis due to an ulcerated plaque is very rare and has not been well reported in the literature. We report an interesting case of 72-year-old man presenting with neurological symptoms secondary to in-stent restenosis of the carotid artery caused by an ulcerated plaque. We also explain the different mechanisms for restenosis along with the treatment options.

## 1. Introduction

Interventional therapy has been highly impacted by the number of lesions treated with stents, which exceeds 50% of all interventional procedures. Although stents have been successful in reducing the restenosis compared to balloon angioplasty, in-stent restenosis (ISR) occurs in 10–60% of cases [1–4]. ISR can be explained by many mechanisms, but restenosis due to an ulcerated plaque is very rare and has not been reported in the literature. We report a case where a patient presented with neurological symptoms secondary to an ulcerated ISR of the carotid artery.

## 2. Case Report

A 72-year-old Caucasian man with past medical history of diabetes mellitus, hypertension, smoking, bilateral carotid endarterectomy, and a left carotid artery stent (14 months ago) presented with symptoms of blurred vision and dizziness. He experienced very similar symptoms prior to the past carotid endarterectomy. A subsequent carotid ultrasound showed a 60–79% stenosis of his left internal carotid artery and no significant stenosis of the right internal carotid artery.

**Figure 1:**
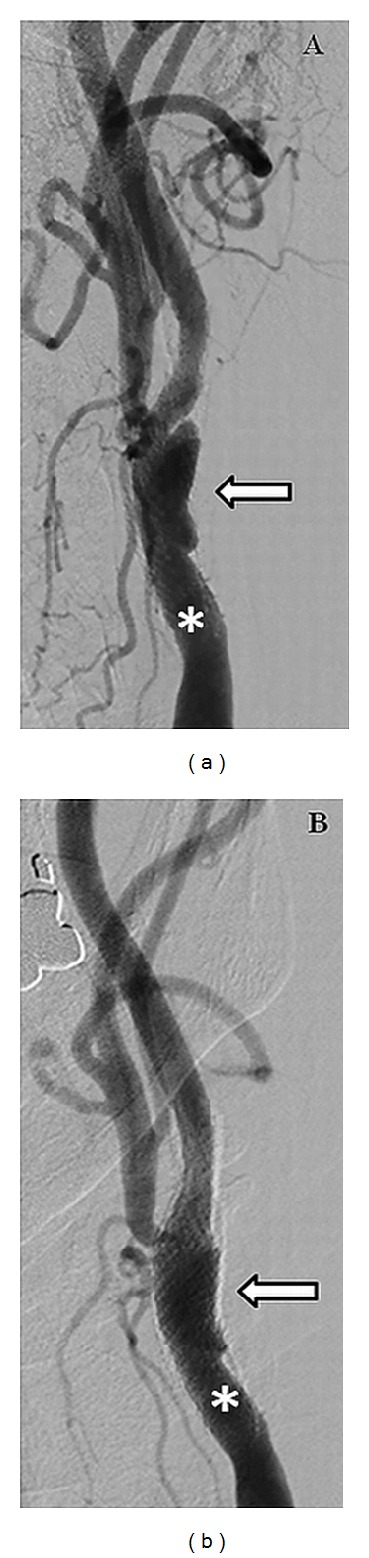
(a) Angiogram of the left carotid artery (asterisk) showing the ulcerated plaque at the stent site resulting in 70% stenosis (arrow). (b) Angiogram of the left carotid artery (asterisk) after the placement of a self-expanding Acculink 7 × 10 × 40 mm stent (arrow).

Carotid angiography showed an eccentric stenosis in the left internal carotid artery at the stent site with a crater/ulcer within the restenosis tissue inside the stent (Figure 1(a)). An Accunet 6.5 filter (Abbott Vascular, Santa Clara, CA) was deployed distal to the stent in the left internal carotid artery. Later, an Acculink (Abbott Vascular) 7 × 10 × 40 mm self-expanding stent was deployed successfully. Postdeployment angiograms revealed brisk flow with no evidence of embolization into the filter, but the ulcerative nature of the crater continued to be present. Balloon dilatation was performed in the mid-portion of the stent after which residual stenosis was approximately 10% with minimal visualization of the crater-like lesion (Figure 1(b)). After successfully retrieving the filter device, final angiograms of cervical and cerebral arteries revealed brisk flow with no evidence of distal embolization maintaining patency of the middle cerebral and anterior cerebral circulation. At the 3-, 6-, 12-, and 24-month follow-up, he was asymptomatic and subsequent carotid ultrasonography did not reveal any restenosis.

## 3. Discussion

In-stent restenosis (ISR) is defined by radiographic findings in CTA (Computed Tomography Angiography), angiography, or duplex. Angiographic ISR is defined by the presence of >50% diameter stenosis in the stented segment [5] and clinically by the presentation of symptoms pertinent to the area supplied by the culprit vessel. Traditionally, ISR has been classified based on the length of the lesion, as focal (<10 mm) or diffuse (>10 mm) [6].

The pathophysiology of in-stent restenosis is neointimal hyperplasia (NIH). Arterial injury after stent placement induces vascular smooth muscle cell proliferation due to a multitude of causes which include (1) mechanical stretch and medial dissection; (2) endothelial denudation resulting in exposure to circulating mitogens like angiotensin II and plasmin; (3) release of cytokines from endothelial cells and platelets. Leukocyte recruitment and platelet accumulation at the site of injury are the hallmarks which initiate restenosis [7]. Endothelial denudation causes polymorphonuclear leukocyte adherence and activation of the platelets. The activated platelets in turn secrete monocyte chemoattractant protein (MCP)-1. Persistently elevated levels of MCP-1 have been documented in patients who develop restenosis [8]. Early phase (days to weeks) of ISR is characterized by the reorganization of thrombus and an acute inflammatory reaction at the stent site. Sustained production of cytokines and adhesion molecules cause further leukocyte recruitment and infiltration. Late phase (weeks to months) of ISR results in phenotypic modification of the medial smooth muscle cells (SMCs), which then migrate and proliferate in the intima [6]. After proliferation, SMCs synthesize extracellular matrix (ECM) which forms up the main bulk of intimal tissue. Neointimal hyperplasia is composed mainly of proteoglycans and collagen with cellular elements making up only about 11% [9]. All the above-mentioned factors act in a regulated fashion and lead to extracellular matrix formation which in turn contributes to NIH and restenosis. These lesions are fibrous in nature and typically resistant to atherosclerosis. In this case our patient had initial stent placed 14 months prior to this episode with good angiographic evidence of the stent placement without any signs of dissection, under deployment of the stent or residual stenosis. Ulceration of restenotic lesion within the stent has not been reported. The uniqueness of this case is that in-stent restenosis underwent typical atherosclerotic degenerative changes leading to formation of a lipid pool which ultimately eroded forming an ulcerated plaque as evident during angiography. It is thought to be due to severe progressive atherosclerosis. Utilization of newer technologies such as intravascular ultrasound (IVUS) and optical coherence tomography (OCT) might have aided in better understanding of the lesion [10, 11]. In this case the operator did not perform the IVUS or OCT.

Management of in-stent restenosis is a well-debated topic and the options include brachytherapy, balloon angioplasty, and restenting. Brachytherapy reduces neointimal proliferation by blocking cell proliferation and inhibiting SMC migration. Unfortunately, it has several after effects, which include late thrombosis and failure of the medial dissection to heal. Balloon angioplasty is a simple and effective treatment for in-stent restenosis, but only for a short period of time. Stenting restenosis is an ideal option and may be an effective long-term solution [12]. The clinical evolution without recurrence of symptoms in 2 years in our case supports this fact.
